# Protective Effects of Pyruvic Acid Salt Against Lithium Toxicity and Oxidative Damage in Human Blood Mononuclear Cells

**DOI:** 10.15171/apb.2019.035

**Published:** 2019-06-01

**Authors:** Evgenii Plotnikov, Innokenty Losenkov, Elena Epimakhova, Nikolay Bohan

**Affiliations:** ^1^Tomsk National Research Medical Center of the Russian Academy of Sciences, Mental Health Research Institute, 634014, Tomsk, Aleutskaya 4, Russia.; ^2^Research School of Chemistry & Applied Biomedical Sciences, Tomsk Polytechnic University, 634050, Tomsk, Lenin av., 30, Russia.

**Keywords:** Apoptosis, Antioxidant, Oxidative stress, Lithium pyruvate, Cytoprotection, Mood-stabilizer

## Abstract

***Purpose:*** Aim of present work was to study cytoprotective properties of lithium pyruvate, as a prospective pharmacological agent. Pyruvate has a lot of potential benefits due to positive influence on cell metabolism. Lithium is "gold-standard" mood-stabilizer. Combination of both may lead advantages.

***Methods:*** Lithium pyruvate was tested as cytoprotector on human blood mononuclears under induced oxidative stress. Cells were obtained from healthy donors and patients with alcoholism. The detection of cell viability, apoptosis and determination of oxidative stress level were conducted by flow cytometry.

***Results:*** Lithium pyruvate showed excellent cytoprotective properties in normal and oxidation conditions. This effect was independent from cell donor health status. It was shown on cells from healthy donors and alcoholics patients.

***Conclusion:*** Obtained results allow considering lithium pyruvate as potential normothymic agents (mood stabilizer) with excellent cytoprotective properties.

## Introduction

Development and study of new effective cytoprotectors is a topical task for biological and medical application. Oxidative stress is a common way which takes part in different pathology.^1-4^ This condition occurs when production of reactive oxygen species (ROS) exceeds cellular defense capabilities. Normally ROS are constantly produced by healthy cells, and antioxidant system regulates redox balance. In some circumstances ROS can damage proteins, nucleic acids, and lipids. Cell antioxidant system includes enzymatic component and low molecular antioxidants. Pyruvic acid is one of them and the key metabolite in aerobic glycolysis. Pyruvate is included in the Krebs cycle via acetylation reaction under the action of acetylcoenzyme A. Pyruvates enhance aerobic endurance in vivo. This is associated with the acceleration, including lipolysis and the increase in the energy-capacity of the cell via Krebs cycle. In addition to a significant energy role, the cycle is also given a significant plastic function, that is, an important source of precursor molecules, from which key compounds for the life of the cell are synthesized, such as amino acids, carbohydrates, fatty acids. It was proved that pyruvate could protect organs and tissue via correction hypoxic lactic acidosis in rats subjected to lethal burn shock.^5^ Pyruvate sodium salt modifies the blood acid-base status and the lactate production during the exercise and improves acidosis resistance.^6^ Some of TCA cycle metabolites can reduce cell death under hypoxic damage.^7^ In general, pyruvate is a highly effective intracellular substrate directly linked to energy metabolism and ATP production. Based on our recent results, pyruvate lithium salt possesses some additional benefits for cytoprotection.

In turn, lithium ions possess neuro-protective properties. Lithium-based drugs are still the gold standard of bipolar affective disorder treatment.^8^ Recent studies suggest that lithium protects neurons from death induced by a wide array of neurotoxic insults, stimulates neurogenesis and could be used to prevent age-related neurodegenerative diseases.

These effects mostly realized through its influences on neuroprotective protein bcl-2 and glycogen synthase kinase 3β in neuronal tissue.^9^ It was established that human neuronal cells cultured in the presence (Li^+^) ions had higher growth rate, more glutathione content, higher Bcl-2 anti-apoptotic protein level and were more resistant to oxidative stress. Commonly, neuroprotective therapeutic mechanisms of lithium, achieved both direct and indirect ways.^10^ Moreover, glucose consumption and glycolytic activity were enhanced in lithium-treated cells and an important release of pyruvate was observed.^11^ In this regards, combination of lithium and pyruvic acid looks promising for possible protective effects regarding against different aspects of oxidative stress and disease metabolic changes.

In this study we investigate influence of lithium pyruvate spontaneous and induced apoptosis in human blood mononuclear cell cultured in vitro. Oxidative stress is one of basic mechanism of apoptosis and cell death. It is especially important due to free radicals may play role in the pathogenesis of mental disorders,^2^ so direct and indirect antioxidant properties may significantly contribute to biological effects of lithium pyruvate in vivo.

However, literature data regarding pro-oxidant and antioxidant effects is rather diverse.^12^ We consider here oxidative stress model on human mononuclear blood cells. Cell response could depend on initial condition and diseases which donors suffer from. Oxidative stress is considered as potential treatment target of psychiatric disorders.^13^ Here we compare lithium effects on blood cells of alcoholics and healthy donor. It was shown previously alcoholism leads to deep exhaustion of blood antioxidant system.^14,15^ Chronic ethanol consumption can induce oxidative stress injury, and neurotransmitter metabolic abnormalities.^16^ Oxidative stress plays a role in the course and complications of alcohol dependence and this explains that the use of antioxidants is an important factor in improving the patient’s somatic state.^15^ Aim of present work was to study cytoprotective properties of lithium pyruvate and compare lithium pyruvate effects on blood cells of healthy donors and patients with alcoholism in condition of induced oxidative stress.

## Materials and Methods

### Chemicals

**Figure 1:**
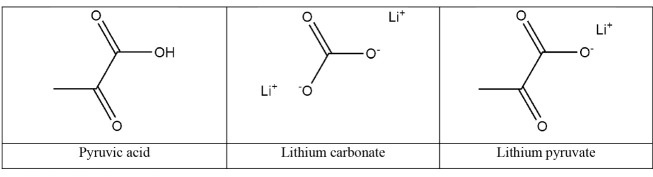
Formulas of investigated lithium salts.

The following lithium compounds (Figure 1) have been used for the researches: lithium pyruvate, lithium carbonate (Sigma-Aldrich, Germany) used as a standard lithium drug. Solution of tert-butyl hydroperoxide (TBHP) 70 wt.% in H_2_O (Sigma-Aldrich, Germany) was used as oxidation inductor to modulate oxidative stress. Lithium pyruvate was prepared for experiments *ex tempore* at Chemical engineering department of Tomsk Polytechnic University. Formulas of all tested substances are shown at Figure 1.

### Human blood mononuclear cells preparation and culturing

The study used blood from 20 healthy control group volunteers, 8 of them men and 12 women aged 25 to 54 years. Peripheral blood mononuclear cells (PBMC) were obtained from whole blood collected in single-use Vacutainer sodium tubes (“Becton Dickinson”, USA) in a sodium heparin tube. Mononuclear cells were isolated from the blood by centrifugation on a ficoll density gradient (ρ = 1.077 g/cm^3^) (Sigma-Aldrich, USA). The resulting cells were resuspended in RPMI 1640 medium. After preparation, the cell suspension was immediately used in the experiment.

### Study of the lithium salts cytoprotective activity

Investigation of the effect of lithium pyruvate on peripheral blood mononuclear cells was carried out using the Annexin V & Dead Cell Assay Kit (Merck Millipore, Germany) on a flow cytometer “Muse Cell Analyzer” (Merck Millipore, Germany). According to the protocol of the study, the cells in the lower left gate of the scattergram were counted as living, in the lower right gate - in the early stage of apoptosis, in the upper right - in the late stage of apoptosis/necrosis, in the upper left gate - dead cells, i.e. state of necrosis.

### Detection of oxidative stress level in cells

The effect of lithium salts on oxidative stress was studied using the “oxidative stress” reagent kit (Merck Millipore, Germany) on a flow cytometer “Muse Cell Analyzer” (Merck Millipore, Germany). According to the protocol, the left peak of the distribution histogram (Gate M1) was counted as the percentage of cells free of reactive oxygen species (ROS-negative), the right peak of the histogram (gate M2) was counted as the percentage of cells containing reactive oxygen species (ROS- positive).

### Cell treatment and maintenance

To assess the cytoprotective activity of lithium salts and the level of induction of oxidative stress in cells, mononuclear cells were incubated in the presence of lithium pyruvate. As a reference, lithium carbonate was used as a normotimic widely used in medicine. All compounds were tested at a therapeutic concentration of 1.2 mmol/L, based on the concentration of lithium ions in the final cell culture. As an inducer of oxidative stress, TBHP was used at a concentration of 50 μm/L. Cells for all experiments were incubated for 24 hours at 37°C in 5% carbon dioxide.

### Statistical analysis

Statistical analysis was performed using the SPSS software, release 20.0. The median, the first and third quartiles were calculated. To compare the quantitative variables, the Mann-Whitney criteria were used. Differences were considered statistically significant at a significance level of *P *< 0.05.

## Results and Discussion

In our previous work we have shown excellent antioxidant properties of some organic lithium salts and Krebs cycle substrates. Pyruvate is one of the perspective antioxidants, positively influence on cell viability and mitochondrial function under stress conditions.^17^ It provides a solid base for further experiment to evaluate influence on cell under oxidation conditions. Percentage of dead cells in culture treated by lithium salts are presented in Table 1. Lithium pyruvate showed significant cytoprotective action with viability level up to 70% in oxidative stress condition. It is very close and comparable to intact cells negative control group. Whereas oxidation control group with TBHP-stressed PBMC without lithium pretreatment showed dramatically drop in cell viability with apoptotic level rise to 80%.

**Table 1:** Lithium salts influence on cell death of PBMC of healthy donors and alcoholics

**Tested substrate**	**Healthy donors cells**			**Cells of patients with alcoholism**		
	**Early apoptosis level. %**	**Late apoptosis/dead cells. %**	**Live cell.%**	**Early apoptosis level. %**	**Late apoptosis/dead cells. %**	**Live cells. %**
Intact cells (control)	12.75 (10.56-15.40)	10.45 (7.90-15.45)	76.4 (70.07-83.47)	8.77 (6.81-16.81)	9.72 (4.75-12.74)	80.4 (72.58-88.53)
Lithium carbonate	11.26 (9.75-13.65)	10.72 (8.35-14.37)	78.42 (73.43-84.44)	7.37 (5.85-11.30)	9.25 (5.03-10.37)	84.7 (77.16-97.98)
Lithium pyruvate	11.25 (9.85-13.84)	9.48 (7.75-12.57)	80.17 (75.61-85.0)	6.87 (4.04-12.55)	6.51 (4.05-10.14)	87.37 (77.95-91.93)
TBHP	31.17 (29.99-32.48)	48.82 (34.19-60.87)	20.42^a^ (8.32-31.98)	37.75 (25.10-40.42)	38.30 (32.30-50.76)	20.70^c^ (10.33-34.30)
TBHP+lithium carbonate	29.39 (23.78-14.53)	51.33 (37.48-56.21)	16.04 (11.35-29.05)	34.15 (29.44-41.02)	44.05 (36.06-47.92)	19.41 (10.79-31.37)
TBHP+lithium pyruvate	18.94 (15.90-23.95)	14.82 (11.55-31.05)	66.56^b^(40.97-72.55)	21.36 (16.70-25.16)	13.25 (11.71-21.12)	63.16^d^ (48.74-70.59)

^a^ *P* < 0.001 compared to intact cells of healthy donors. ^b^ *P* = 0.001 compared to cells of healthy donors incubated with TBHP alone. ^c^ *P* < 0.001 compared to intact cells of patients with alcoholism. ^d^ *P* = 0.003 compared to cells of healthy donors incubated with TBHP alone. Note: Cells were cultured under normal and induced oxidative condition (n=20), Mann-Whitney test, presented as Мedian value (Q1 – Q3).

A cytotoprotective effect of lithium pyruvate was found, consisting of a statistically significant (*P *< 0.05) decrease in the percentage of cells with early apoptosis and late apoptosis/necrosis and an increase in the number of viable cells during incubation under induced oxidative stress. Obtained results clearly showed expressed cytoprotective effect of lithium pyruvate on human cells under induced oxidation stress. This effect appears independently of donor’s health status. Similar action was observed for blood cells from patients suffering from alcoholism (Table 1). Moreover, moderate cytoprotective actions of lithium pyruvate were detectable at normal medium condition without oxidation agent (TBHP).

We evaluate changes in ROS in cell to revealed mechanism of cell death induction. Prevention of oxidation damage was shown in lithium pyruvate treated cells (Table 2).

**Table 2:** Oxidation stress level (cells with ROS, %) under the action of lithium salts on peripheral blood mononuclear cells (n = 20) incubated in the presence of tert-butyl hydroperoxide (TBHP)

	**Healthy donors cells ROS-positive. %**	**Alcoholics cells. ROS-positive. %**
Intact cells (control)	11.03 (7.93-15.53)	7.08 (5.26-13.38)
Lithium carbonate	8.37 (6.64-12.64)	5.50 (5.09-8.83)
Lithium pyruvate	6.53^a^(5.58-9.96)	4.93^b^ (4.42-5.47)
TBHP	65.33^c^ (41.95-79.30)	61.33^d^ (51.52-73.43)
TBHP+lithium carbonate	69.55 (36.48-82.02)	61.08 (52.73-78.25)
TBHP+lithium pyruvate	42.70^e^ (16.73-58.70)	24.53^f^ (14.67-29.98)

^a^ *P* = 0.009 compared to intact cells of healthy donors. ^b^ *P* = 0.005 compared to intact cells of patients with alcoholism. ^c^ *P* < 0.001 compared to intact cells of healthy donors. ^d^ *P* < 0.001 compared to intact cells of patients with alcoholism. ^e^ *P* < 0.001 compared to cells of healthy donors incubated with TBHP alone. ^f^ *P* < 0.001 compared to cells of patients with alcoholism incubated with TBHP alone. Note: Mann-Whitney test, presented as Мedian value (Q1 – Q3).

As it shown in Table 2, apoptotic level and ROS status of the cells directly correlated. It was revealed decrease of ROS in cells with lithium; meanwhile in positive TBHP-treated control more than 90% cells were ROS-positive. Under conditions of sufficient oxygen supply, the pyruvic acid is converted to acetyl coenzyme A and further the cascade of transformations along the Krebs cycle. Pyruvate can also be converted into an anaplerotic reaction into oxaloacetate. In this context, the effect of pyruvic acid and its salts on the key respiratory process of all cells is evident. Due to extreme complexity of metabolic interactions, pyruvate influence on many intracellular processes directly or indirectly. The Krebs cycle is the center of the intersection of many metabolic pathways in the body, an intermediate stage between glycolysis and the electron transport chain. Lithium has additional influence on cell resistance in this case. It has been elucidated inter-associations between GSK3β-mediated antioxidative and neurotransmission mechanisms.^18^ Oxidative stress level in cells was considered as main mechanism of apoptosis induction. Recent work showed excellent antioxidant activity of some organic lithium salt.^19^ It depends mostly of anionic component. Lithium can change of intracellular pathways of apoptosis due to important role in mitochondrial function via inositol depletion.^20^

As can be seen from Table 2, lithium pyruvate possessed a pronounced antioxidant effect, statistically significant (*P*<0.001) decreasing the content of cells with active forms of oxygen. The distribution of cells by the expression level of the radicals under the influence of lithium pyruvate does not differ from the intact control. This notable cytoprotective effect could be partly explained by direct antioxidant action of pyruvate, that primarily decrease level of ROS. Another important contribution is pyruvate influence on energetic metabolism and ATP production that eventually increase cell resistance. Previously was shown that pyruvate effectively protected red blood cells during cardiopulmonary bypass procedure.^21^ Thus, despite of different damage factors, protective effect is common. The study of the level of oxidative stress in cell culture is completely correlated with the level of cytotoxicity and both indicators are identically reduced in the presence of lithium pyruvate.

Lithium pyruvate act as antioxidant and significantly influences on cell metabolism, changing intracellular regulation leading significant anti-apoptotic effect.

## Conclusion

A pronounced cytoprotective effect of lithium pyruvate was established at oxidative stress with respect to peripheral human blood mononuclear cells. The protective effect of pyruvate manifests regardless of the somatic state of the donor cells and the presence of diseases, in particular alcoholism. This effect is mainly due to the direct antioxidant effect of pyruvate and its beneficial influence on intracellular energy processes. Lithium also possess an anti-apoptotic action, but its effect is less expressed here. Lithium pyruvate and other salts based on Krebs cycle substrates could be considered as prospective psychotropic antioxidants for further experiments in vivo.^22^

## Ethical Issues

Not applicable.

## Conflict of Interest

The authors declare no conflict of interest.

## Acknowledgments

This work was supported by the Russian Science Foundation (Project No. 17-75-20045).
